# Tripartite motif-containing protein 46 accelerates influenza A H7N9 virus infection by promoting K48-linked ubiquitination of TBK1

**DOI:** 10.1186/s12985-022-01907-x

**Published:** 2022-11-03

**Authors:** Wei Su, Xian-Tian Lin, Shuai Zhao, Xiao-Qin Zheng, Yu-Qing Zhou, Lan-Lan Xiao, Hui Chen, Zheng-Yu Zhang, Li-Jun Zhang, Xiao-Xin Wu

**Affiliations:** 1 Department of Intensive Care Unit, Guangzhou First People’s Hospital, Guangzhou Medical University, Guangzhou, 510180 Guangdong China; 2 State Key Laboratory for Diagnosis and Treatment of Infectious Diseases, National Clinical Research Centre for Infectious Diseases, Collaborative Innovation Center for Diagnosis and Treatment of Infectious Diseases, The First Affiliated Hospital, Zhejiang University School of Medicine, 79 Qing Chun Road, Hangzhou, 310003 Zhejiang China; 3 Department of Lung Transplant, The Second Affiliated Hospital, Zhejiang University School of Medicine, Hangzhou, 310003 Zhejiang China; 4 Department of Respiratory, Sir Run Run Shaw Hospital, Zhejiang University School of Medicine, Hangzhou, 310020 Zhejiang China

**Keywords:** Influenza A H7N9, TRIM46, TBK1, Interferons

## Abstract

**Background:**

Avian influenza A H7N9 emerged in 2013, threatening public health and causing acute respiratory distress syndrome, and even death, in the human population. However, the underlying mechanism by which H7N9 virus causes human infection remains elusive.

**Methods:**

Herein, we infected A549 cells with H7N9 virus for different times and assessed tripartite motif-containing protein 46 (TRIM46) expression. To determine the role of TRIM46 in H7N9 infection, we applied lentivirus-based TRIM46 short hairpin RNA sequences and overexpression plasmids to explore virus replication, and changes in type I interferons and interferon regulatory factor 3 (IRF3) phosphorylation levels in response to silencing and overexpression of TRIM46. Finally, we used Co-immunoprecipitation and ubiquitination assays to examine the mechanism by which TRIM46 mediated the activity of TANK-binding kinase 1 (TBK1).

**Results:**

Type I interferons play an important role in defending virus infection. Here, we found that TRIM46 levels were significantly increased during H7N9 virus infection. Furthermore, TRIM46 knockdown inhibited H7N9 virus replication compared to that in the control group, while the production of type I interferons increased. Meanwhile, overexpression of TRIM46 promoted H7N9 virus replication and decrease the production of type I interferons. In addition, the level of phosphorylated IRF3, an important interferon regulatory factor, was increased in TRIM46-silenced cells, but decreased in TRIM46 overexpressing cells. Mechanistically, we observed that TRIM46 could interact with TBK1 to induce its K48-linked ubiquitination, which promoted H7N9 virus infection.

**Conclusion:**

Our results suggest that TRIM46 negatively regulates the human innate immune response against H7N9 virus infection.

**Supplementary Information:**

The online version contains supplementary material available at 10.1186/s12985-022-01907-x.

## Background

The influenza A virus, avian influenza H7N9 virus, belongs to the *Orthomyxoviridae* family. H7N9 virus emerged in China in 2013 and posed a threat to public health [1–3]. As we all know, the H7N9 influenza viruses have caused over 1500 human infections, with a mortality rate of nearly 40%. A number of previous studies have offered valuable information on the pathogenesis, prevention and control of the H7N9 virus. [4–7]. Most patients infected with H7N9 developed acute respiratory distress syndrome (ARDS) and severe lung pneumonia, which was caused by a fierce increase in the expression levels of cytokines and chemokines [2, 8]. Wan et al. [9] found that a ‘cytokine storm’ in the lungs of H7N9-infected patients was associated with activation of gasdermin E (GSDME)-mediated pyroptosis in alveolar epithelial cells. However, the host factors involved in viral replication remains elusive. Thus, a better understanding of the regulatory mechanisms of H7N9 infection would be useful to combat future H7N9 virus outbreaks.

The first line of defense against invading pathogens is the innate immune response. Pattern recognition receptors (PRRs) recognize pathogen-associated molecular patterns, which subsequently activates the downstream innate immune response [10, 11]. During influenza virus infection, the RIG-I (retinoic acid inducible gene I) receptor senses influenza genomic RNA and recruits the mitochondrial antiviral signaling protein (MAVS) and TANK-binding kinase 1 (TBK1) to induce the phosphorylation, dimerization, and nuclear translocation of interferon regulatory factor 3 (IRF3), which finally induces the production of type I interferons [12–14].

The tripartite motif family (TRIM) of proteins have been intensively studied in virus infection. One member, TRIM46, could regulate cancer cell viability, apoptosis, and the cell cycle [15–17]. However, the function of TRIM46 in H7N9 infection and its underlying mechanism remain to be determined. In this study, we aimed to identify the function of TRIM46 in H7N9 virus infection and the underlying mechanism between TRIM46 and the production of host RLR-dependent type I interferons. The results showed that, during H7N9 virus infection, TRIM46 acts as a negative regulator of the host innate immune response. Upon H7N9 virus infection, TRIM46 expression gradually increased over time. Furthermore, knockdown of TRIM46 resulted in increased production of type I interferons and phosphorylation of IRF3, whereas its overexpression had the opposite effects. Finally, we observed that TRIM46-mediated K48-linked ubiquitination of TBK1 resulted in the inhibition of host innate immunity. Thus, this study revealed novel activities of TRIM46 in innate immunity, which potentiates the study of innate immunity against virus infection.

## Methods

### Cell culture and virus strain

The American Type Culture Collection (ATCC, Manassas, VA, USA) provided the A549, HEK293T, and Madin-Darby canine kidney (MDCK) cells. The cells were grown in Dulbecco’s modified Eagle medium supplemented with heat-inactivated 10% fetal bovine serum, 1% penicillin (100 U/mL), and streptomycin sulfate (100 mg/mL). All cell lines were cultured in a 37 °C incubator with an atmosphere of 5% CO_2_. Ten-day-old embryonated specific-pathogen free chicken eggs were used to isolate and propagate Influenza A Virus strain A/Zhejiang/DTID-ZJU01/2013(H7N9). The allantoic fluid from the infected chicken eggs was collected and preserved at − 80 °C. The median tissue culture infectious dose (TCID_50_) method was used to determine the virus titer, which was calculated using the Reed-Muench method. All the live H7N9 virus experiments were performed in a bio-safety level 3 laboratory at the First Affiliated Hospital, Zhejiang University School of Medicine (Registration No. CNAS BL0022).

### Lentivirus-mediated plasmid transfection

For TRIM46 knockdown, two short hairpin interfering (shRNA) sequences were designed against two different regions of TRIM46 (the target sequence of TRIM46#1 was 5’- GCTGCTGACAGAGCTTAACTT -3’, the target sequence of TRIM46#2 was 5’- CTGGCACTATACCGTTGAGTT -3’) and cloned and packed into lentiviruses. A TRIM46 overexpression construct was also created and cloned and packed into lentiviruses. The TRIM46 shRNA and overexpression lentiviruses were transfected separately into A549 cells for 72 h. The transfection efficiency was observed using a fluorescence microscope (Olympus, Tokyo, Japan).

### Western blotting analysis

Cells were harvested and lysed for 30 min in radioimmunoprecipitation assay (RIPA) buffer with phenylmethylsulfonyl fluoride (PMSF) and phosphatase inhibitors. The lysed cells were then subjected to centrifugation for 10 min at 12,000 × rpm and 4 °C. We retained the supernatants and determined their protein contents using a bicinchoninic acid protein assay. Equal amounts of proteins were subjected to 12% sodium dodecyl sulfate-polyacrylamide gel electrophoresis separation. The separated proteins were electrotransferred onto a polyvinyl difluoride membrane. Non-specific binding was blocked by incubating the membranes in 5% skim milk in Tris-buffered saline-Tween 20 (TBST) at room temperature for 1 h. The membranes were then added with the appropriate primary antibodies and incubated overnight at 4 °C. Next day, three washes with TBST carried out and then the membranes were incubated with the corresponding horseradish peroxidase (HRP)-conjugated secondary antibodies. An enhanced chemiluminescence (ECL) reagent was used to visualize the immune-reactive proteins. Primary antibodies against TRIM46 (ab169044), Influenza A nucleoprotein (NP) (ab128193), Myc tag (ab9106), and glyceraldehyde-3-phosphate dehydrogenase (GAPDH) (ab8245) were purchased from Abcam (Cambridge, UK). Primary antibodies against phosphorylated (p)-IRF3 (#4947) and IRF3 (#4302) were provided by Cell Signaling Technology, Inc. (Danvers, MA, USA). Sigma-Aldrich (Darmstadt, Germany) provided the anti-FLAG Tag antibody.

### Quantitative real-time reverse transcription PCR (qRT-PCR)

The TRIzol reagent (Invitrogen, Waltham, MA, USA) was used to extract total RNA from cells. Reverse transcription was then used to produce cDNA from the total RNA.

For influenza virus replication, NP RNAs were reverse-transcribed with primers as following: NP mRNA using oligo (dT), NP cRNA using 5’- AGTAGAAACAAGG -3’, NP vRNA using 5’- AGCGAAAGCAGG -3’. The cDNA was then quantified using quantitative real-time PCR with gene-specific primers. *GAPDH* mRNA was quantified as an internal control and the 2^−ΔΔCt^ method was used to analyze the relative quantity of the target genes. The primers used in this study were as follows:

*TRIM46*

Forward primer: 5ʹ-GATTGCCCGAGCCACTGAA-3ʹ,

Reverse primer: 5ʹ-AGGCACTCGCAGGAAGTTAAG-3ʹ;

*NP*

Forward primer: 5ʹ-ATCAGACCGAACGAGAATCCAGC-3ʹ,

Reverse primer: 5ʹ-GGAGGCCCTCTGTTGATTAGTGT-3ʹ;

*IFNA (encoding interferon alpha)*

Forward primer: 5ʹ-GCCTCGCCCTTTGCTTTACT-3ʹ,

Reverse primer: 5ʹ-CTGTGGGTCTCAGGGAGATCA-3ʹ;

*IFNB1 (encoding interferon B1)*

Forward primer: 5ʹ-AAAGAAGCAGCAATTTTCAGC-3ʹ,

Reverse primer: 5ʹ-CCTTGGCCTTCAGGTAATGCA-3ʹ;

*GAPDH*

Forward primer: 5ʹ-AGGTGAAGGTCGGAGTCA-3ʹ,

Reverse primer: 5ʹ-GGTCATTGATGGCAACAA-3ʹ.

### Co-immunoprecipitation (Co-IP)

The indicated plasmids were transfected into HEK293T cells. The cells were collected and lysed at 4 °C for 30 min in IP lysis buffer (1% NP-40, 0.025 M Tris-HCl, 0.15 M NaCl, 1 mM EDTA, 5% glycerol) supplemented with phosphatase inhibitor/PMSF, followed by centrifugation for 10 min at 12,000 × rpm under 4 °C. We retained the supernatants, one third of which was used for input analysis and the other two thirds were incubated with Anti-Myc Magnetic beads (Pierce #88842, Thermo Fisher Scientific, Waltham, MA, USA), or IgG control, overnight at 4 °C for IP analysis. IP lysis buffer was then used to wash the precipitates three times, followed by boiling the samples in 2 × loading buffer. Western blotting was then used to analyze the precipitates using the indicated primary antibodies, followed by incubation with HRP-conjugated anti-rabbit IgG (conformation specific) (#5127, Cell Signaling Technology) or anti-mouse IgG (Light Chain Specific) (#58,802, Cell Signaling Technology) secondary antibodies. The immune-reactive proteins were visualized using the ECL reagent.

### Ubiquitination assay

We transfected HEK293T cells with a TBK1-Flag plasmid (overexpressing FLAG-tagged TBK1) together with the TRIM46-Myc plasmid (overexpressing Myc-tagged TRIM46) or not. The cells were harvested at 24 h after transfection and lysed in 1% SDS buffer (50 mM Tris (pH 8.1), 1% SDS, sodium pyrophosphate, EDTA) supplemented with a protease inhibitor cocktail at 4 °C for 30 min. The cell extracts were then subjected to IP using anti-Flag magnetic beads at 4 °C overnight. The beads were washed three times and then subjected to western blotting using antibodies recognizing wild-type (WT) Ubiquitin (ab134953), K48-linked Ubiquitin (ab140601), and K63-linked Ubiquitin (ab179434) (all from Abcam).

### Statistical analysis

Data analysis and processing were carried out using GraphPad Prism software version 7 (GraphPad Inc., La Jolla, CA, USA). The statistical difference between two groups was analyzed using an unpaired Student’s *t*-test and one-way analysis of variance (ANOVA) was carried out to analyze the differences among multiple groups. Statistical significance was indicated by *p* < 0.05. In all figures, * indicates *p* < 0.05, ** indicates *p* < 0.01, and *** indicates *p* < 0.001.

## Results

### TRIM46 expression is upregulated in H7N9 virus-infected A549 cells.

**Fig. 1:**
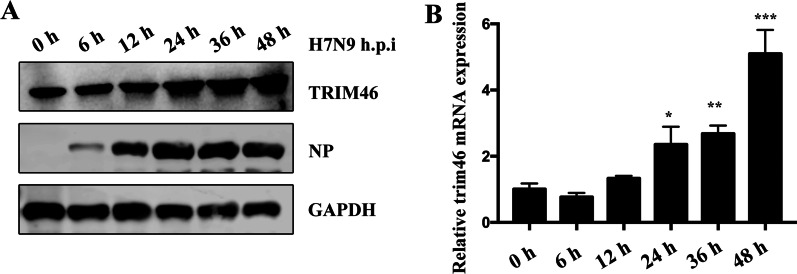
H7N9/ZJU-1 increases TRIM46 expression. A549 cells were seeded in 6-well plate and infected with H7N9/ZJU-1 for indicated times, cells were harvested. The protein expression levels of TRIM46, H7N9/ZJU-1 NP were measured by western blotting and GAPDH was used as an internal control (**A**), and the relative mRNA of TRIM46 at indicated times were analyzed by RT-qPCR (**B**)

First, to determine the relationship between H7N9 virus infection and TRIM46 expression, we infected the human lung adenocarcinoma epithelial cell line A549 with H7N9 virus for different times and detected the TRIM46 protein level using western blotting and the *TRIM46* mRNA expression level using qRT-PCR. The results showed that H7N9 virus induced the TRIM46 protein at different times, reaching a peak at 48 h post infection (h.p.i) (Fig. 1A). The mRNA level increased gradually, also peaked at 48 h.p.i. (Fig. 1B). These results indicated the H7N9 virus infection could induce TRIM46 expression.

### TRIM46 knockdown inhibits H7N9 virus infection

**Fig. 2:**
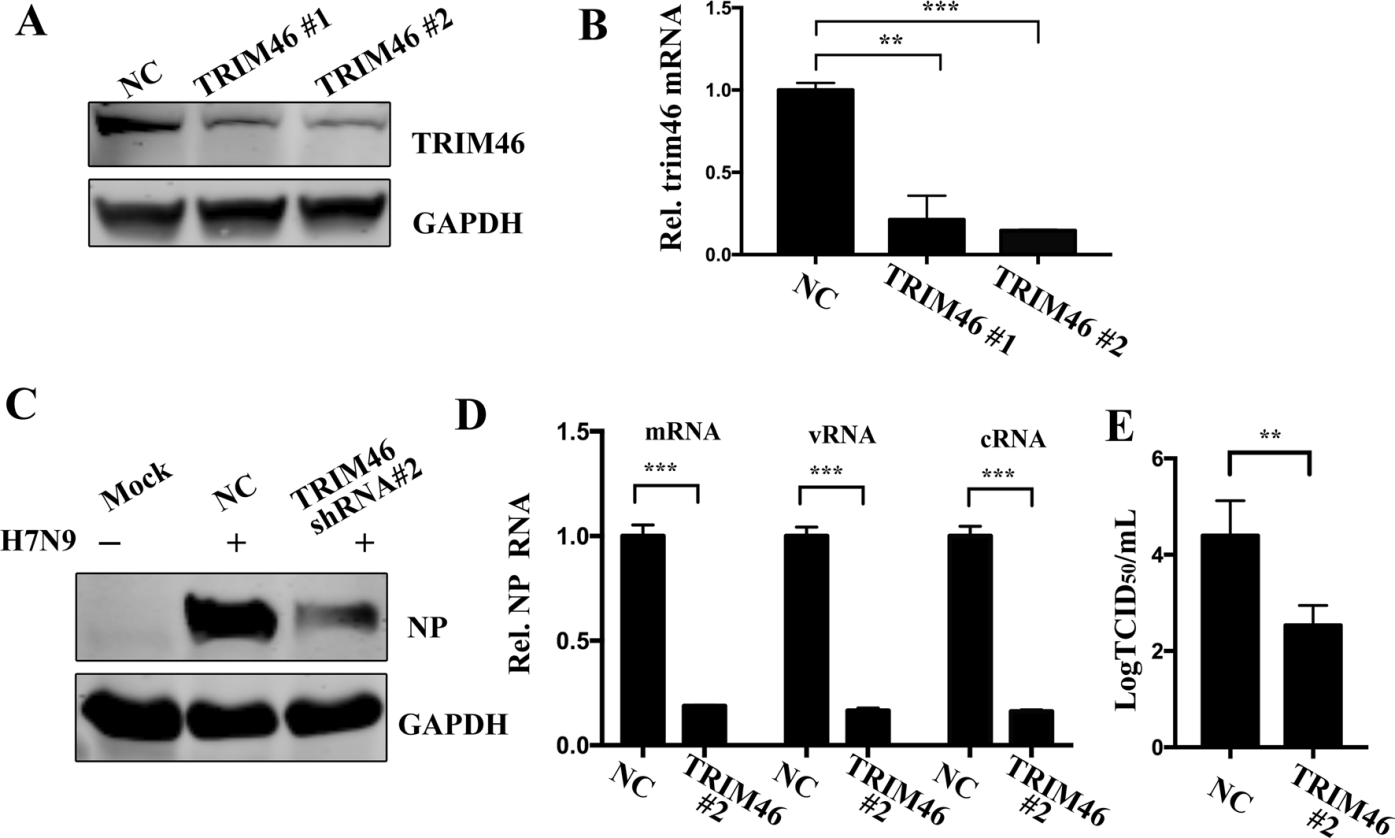
Knockdown of TRIM46 inhibits H7N9/ZJU-1 replication. A549 cells were transfected with negative control sequences and TRIM46 knockdown sequences, TRIM46#1 and TRIM46#2, for 72 h, cells were harvested, the protein levels of TRIM46 were measured by western blotting (**A**) and the mRNA levels were measured by RT-qPCR (**B**). **C**. A549 cells were transfected with TRIM46 shRNA sequence or negative control sequences for 72 h, infected with H7N9/ZJU-1 for 12 h, the cells were lysed and subjected to western blotting with indicated antibodies. TRIM46 Knockdown cells and negative control cells were infected with H7N9/ZJU-1 for 12 h, the relative levels of NP mRNA, cRNA and vRNA were measured by RT-qPCR (**D**). The supernatant of TRIM46 knockdown cells and negative control cells was collected and viral titers were determined by TCID_50_ method (**E**)

To explore whether TRIM46 expression could regulate H7N9 virus infection, we used lentivirus-packaged TRIM46 shRNA#1 and TRIM46 shRNA#2 sequences to generate A549 TRIM46 knockdown cells. We then detected the protein and mRNA levels of TRIM46. The results showed that TRIM46 shRNA#1and #2 both worked well to reduce the protein and mRNA levels of TRIM46 in A549 cells (Fig. 2A, B). A549 cells transfected with negative control or TRIM46 knockdown cells were infected with H7N9 virus for 12 h and A549 mock group cells were used as a control group. The results showed that TRIM46 knockdown significantly decreased the expression of the H7N9 virus NP protein (Fig. 2C). The qRT-PCR showed decreased NP mRNA, cRNA and vRNA expression (Fig. 2D) and the TICD_50_ detection of the virus titer showed that TRIM46 knockdown decreased the H7N9 virus titer compared with that in the negative control group (Fig. 2E). Taken together, the results showed that knockdown of TRIM46 reduced H7N9 virus replication.

### TRIM46 overexpression promoted H7N9 virus replication

**Fig. 3:**
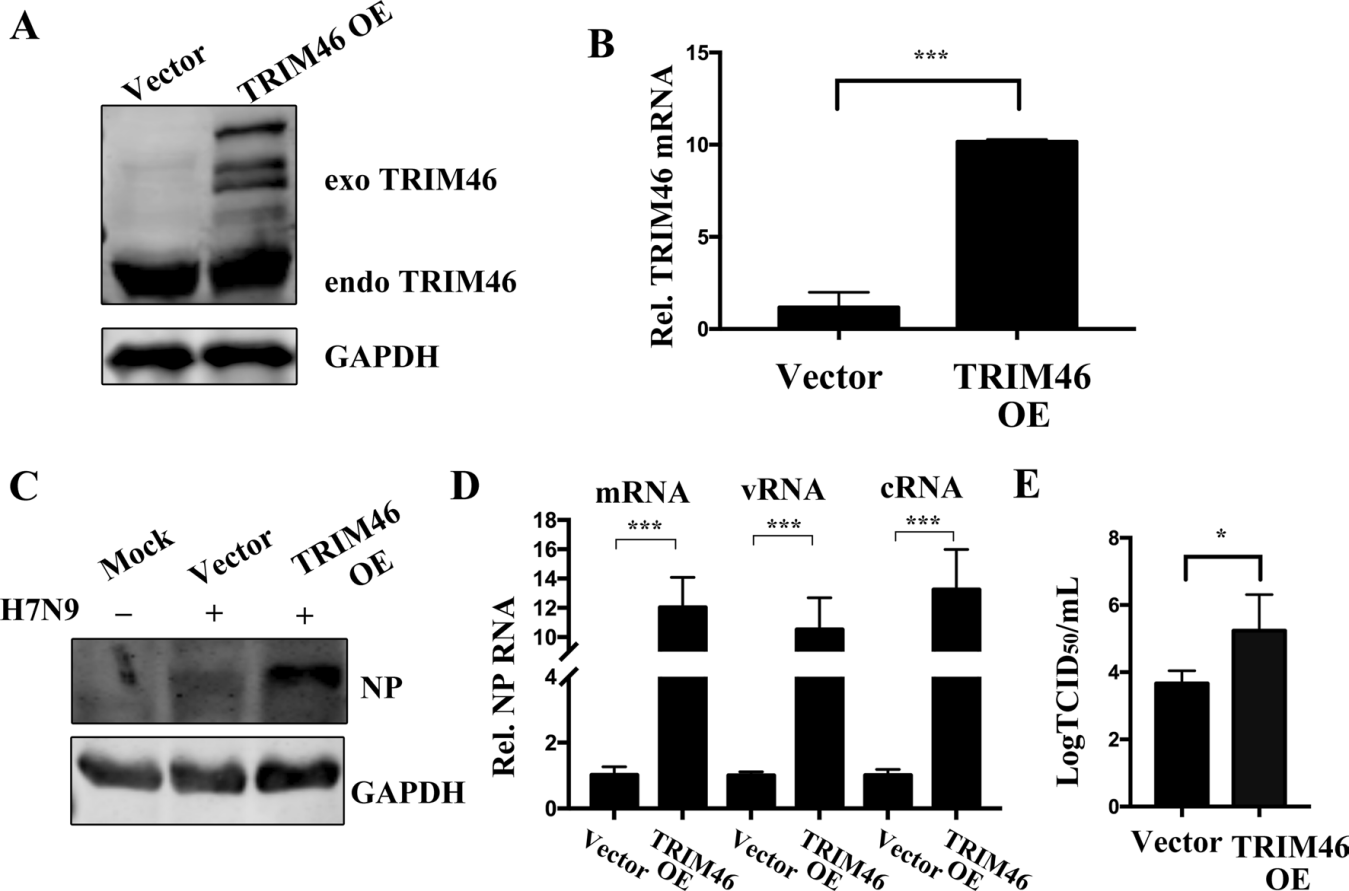
Overexpression of TRIM46 promotes H7N9/ZJU-1 infection. **A.** A549 cells were transfected with lentivirus-mediated TRIM46-Myc plasmids or empty vector plasmids, after 72 h transfection, the cells were harvested and subjected to western blotting for TRIM46 overexpression analysis, GAPDH was used as an internal control. **B**. A549 cells transfected with lentivirus-mediated TRIM46-Myc plasmids or empty vector plasmids for 72 h, A549 cells were harvested and the relative levels of TRIM46 mRNA were analyzed by RT-qPCR. **C**. A549 cells were transfected with empty vector plasmids or TRIM46-Myc plasmids, after 72 h, A549 cells were infected with H7N9 /ZJU-1 (MOI = 1) or mock-treated for 12 h, the cells lysates were collected and subjected to western blotting with indicated antibodies. **D**. TRIM46-Myc overexpression A549 cells or empty vector-transfected A549 cells were infected with H7N9/ZJU-1 (MOI = 1) for 12 h, the relative levels of NP mRNA, cRNA and vRNA were analyzed by RT-qPCR. E. A549 cells were transfected with lentivirus-mediated TRIM46-Myc plasmids or empty vector plasmids, after 72 h transfection, the cells were infected with H7N9/ZJU-1 for 12 h, the supernatant was collected and the viral titers were determined by TCID_50_ method. The analysis results were presented with mean ± SD, in all situations, a *p* value < 0.05 was considered statistically significant, *p < 0.05, **p < 0.01, ***p < 0.001

We further determined whether overexpression of TRIM46 could promote H7N9 virus replication. We transfected A549 cells with lentivirus-packaged empty vector or TRIM46 overexpression plasmids for 48 h, and then used western blotting and qRT-PCR to detect the protein and mRNA levels of TRIM46. The results showed successful overexpression of TRIM46 in A549 cells (Fig. 3A, B). Subsequently, we infected the A549 empty vector group and TRIM46 overexpression group with H7N9 virus for 12 h, and then detected the NP protein and mRNA, cRNA and vRNA expression levels and the H7N9 virus titer. The results showed that overexpression of TRIM46 increased the H7N9 virus NP protein and RNA levels and increased the H7N9 virus titer compared with that of the empty vector group (Fig. 3C–E).

### TRIM46 negatively regulates type I IFNs and the activation of IRF3

**Fig. 4:**
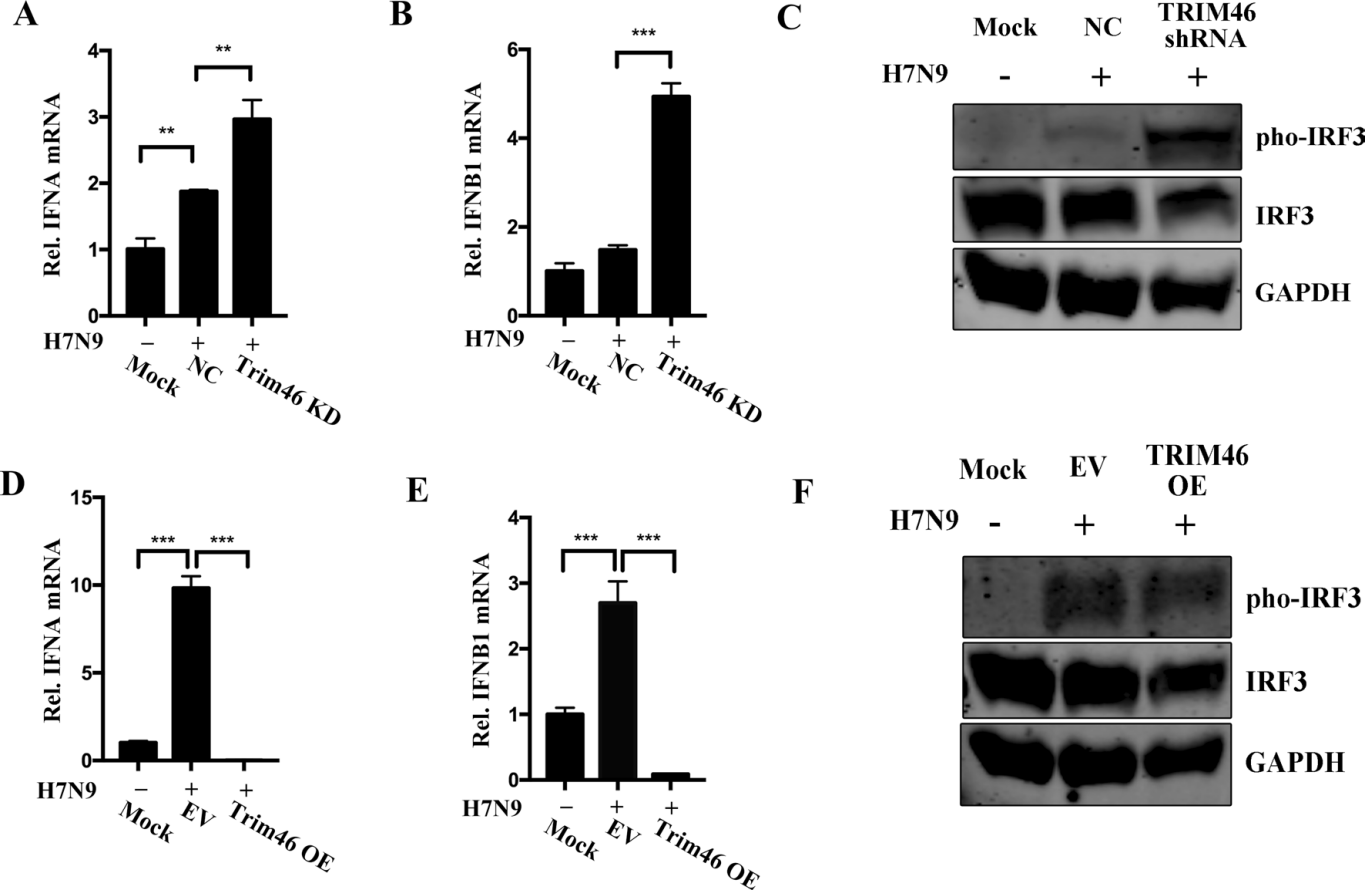
**A**–**C** Knockdown of TRIM46 accelerates H7N9/ZJU-1-induced type I interferon expression. A549 cells were transfected with lentivirus-mediated TRIM46 knockdown sequences or negative control sequences, after 72 h, the cells were infected with H7N9/ZJU-1 or mock-treated for 12 h, the cells were harvested and subjected to RT-qPCR for analysis of IFNA mRNA (**A**) and IFNB1 mRNA (**B**). A549 cells were transfected with lentivirus-mediated TRIM46 shRNA sequence for 72 h, infected with H7N9/ZJU-1 (MOI = 1) for another 12 h. The cells were lysed and pho-IRF3, IRF3 and GAPDH were measured by western blotting (**C**). **D**–**F** Overexpression of TRIM46 inhibits H7N9/ZJU-1-induced type I interferon expression. A549 cells were transfected with TRIM46-Myc endogenous plasmids or empty vector plasmids for 72 h, cells were infected with H7N9/ZJU-1 or mock-infected for 12 h, cells were collected and subjected to RT-qPCR for analyzing relative levels of IFNA mRNA (**D**) and IFNB1 mRNA (**E**). A549 cells were transfected with TRIM46-Myc expression plasmids or empty vector plasmids for 72 h, thereafter, infected with H7N9/ZJU-1 (MOI = 1). Cells lysates were collected and subjected to western blotting with anti-pho-IRF3, IRF3 and GAPDH antibodies (**F**). The results were shown as mean ± SD, **p < 0.01, ***p < 0.001

Type I IFNs plays an important role in defending against virus infection. To examine the role of TRIM46 in the context of viral infection, we examined the expression levels of *IFNA* and *IFNB1*. Knockdown of TRIM46 increased *IFNA* and *IFNB1* mRNA expression levels during influenza H7N9 infection (Fig. 4A, B), meanwhile, ectopic expression of TRIM46 decreased *IFNA* and *IFNB1* mRNA levels (Fig. 4D, E). IRF3 activation is essential for the production of type I IFNs during virus infection. Therefore, we determined the level of phosphorylated IRF3 in influenza H7N9-infected cells. The results showed that TRIM46 knockdown increased the level of phosphorylated IRF3 and overexpression of TRIM46 decreased the level of phosphorylated IRF3 (Fig. 4C, F).

### TRIM46 interacts with TBK1

**Fig. 5:**
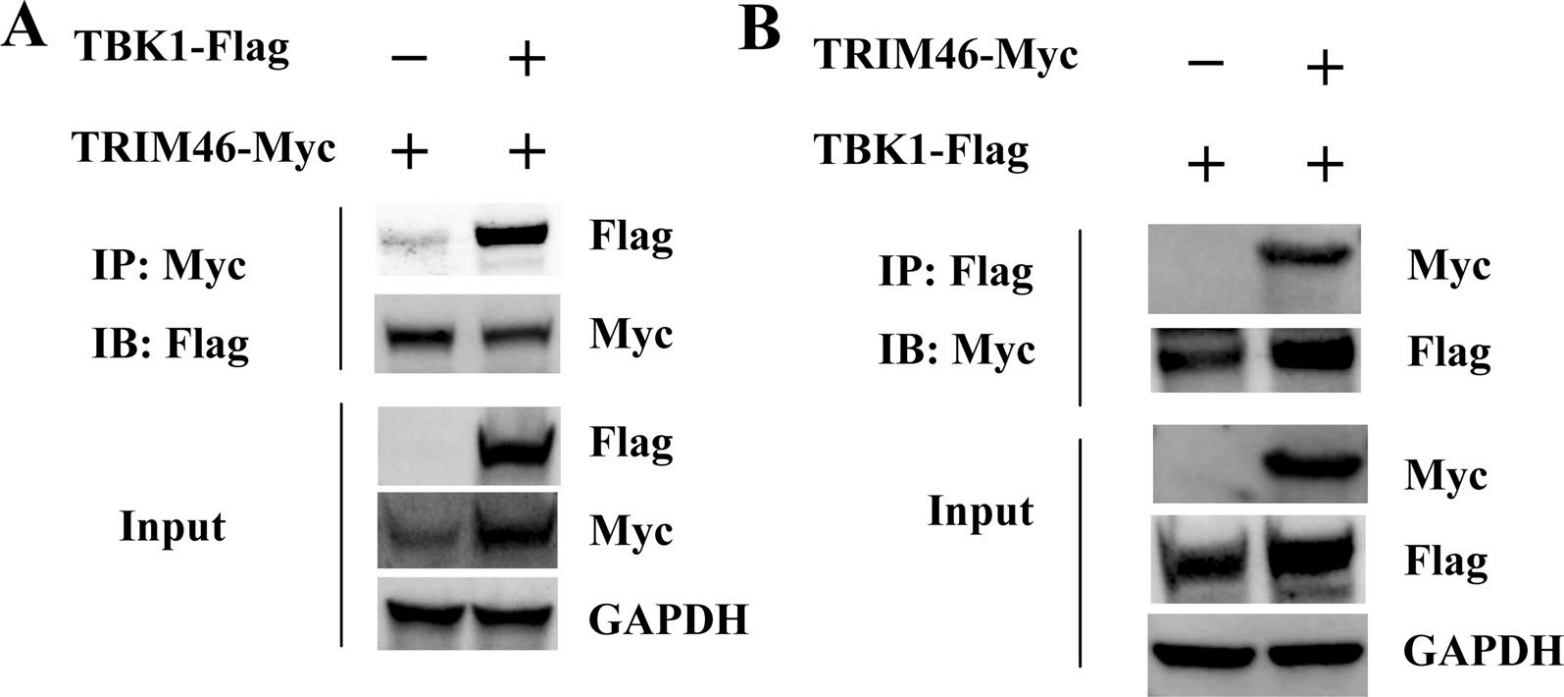
TRIM46 interacts with TBK1. **A**. HEK293T cells were transfected with TRIM46-Myc plasmids with or not with TBK1-FLAG plasmids. After 24 h transfection, the cells were lysed and immuno-precipitated with anti-Myc antibody and subjected to western blotting with anti-Myc and anti-FLAG antibodies. The input cell lysates were analyzed by western blotting with anti-Myc and anti-FLAG antibodies, and GAPDH was used as an internal control. **B**. HEK293T cells were transfected with empty vector plasmids or TRIM46-Myc expression plasmids for 24 h, the cells were lysed and immuno-precipitated with anti-Flag antibody and subjected to western blotting with anti-Myc or anti-Flag antibodies. Whole cell lysates were subjected to western blotting with anti-Myc and anti-Flag antibodies and GAPDH was used as an internal control. All data were repeated for three independent experiments and the data shown as one representative of the triplicate experiments

To investigate the interaction between TRIM46 and TBK1, TRIM46-Myc overexpression plasmids, together with TBK1-Flag or empty vector were transfected into HEK293T cells for 24 h, followed by a Co-IP. As expected, the results showed that TRIM46-Myc interacted with TBK1-Flag (Fig. 5, Additional file 1: Fig. 1A). The above results suggested that TRIM46 interacts with TBK1 to regulate innate immune response.

### TRIM46 promotes K48-linked ubiquitination of TBK1

**Fig. 6:**
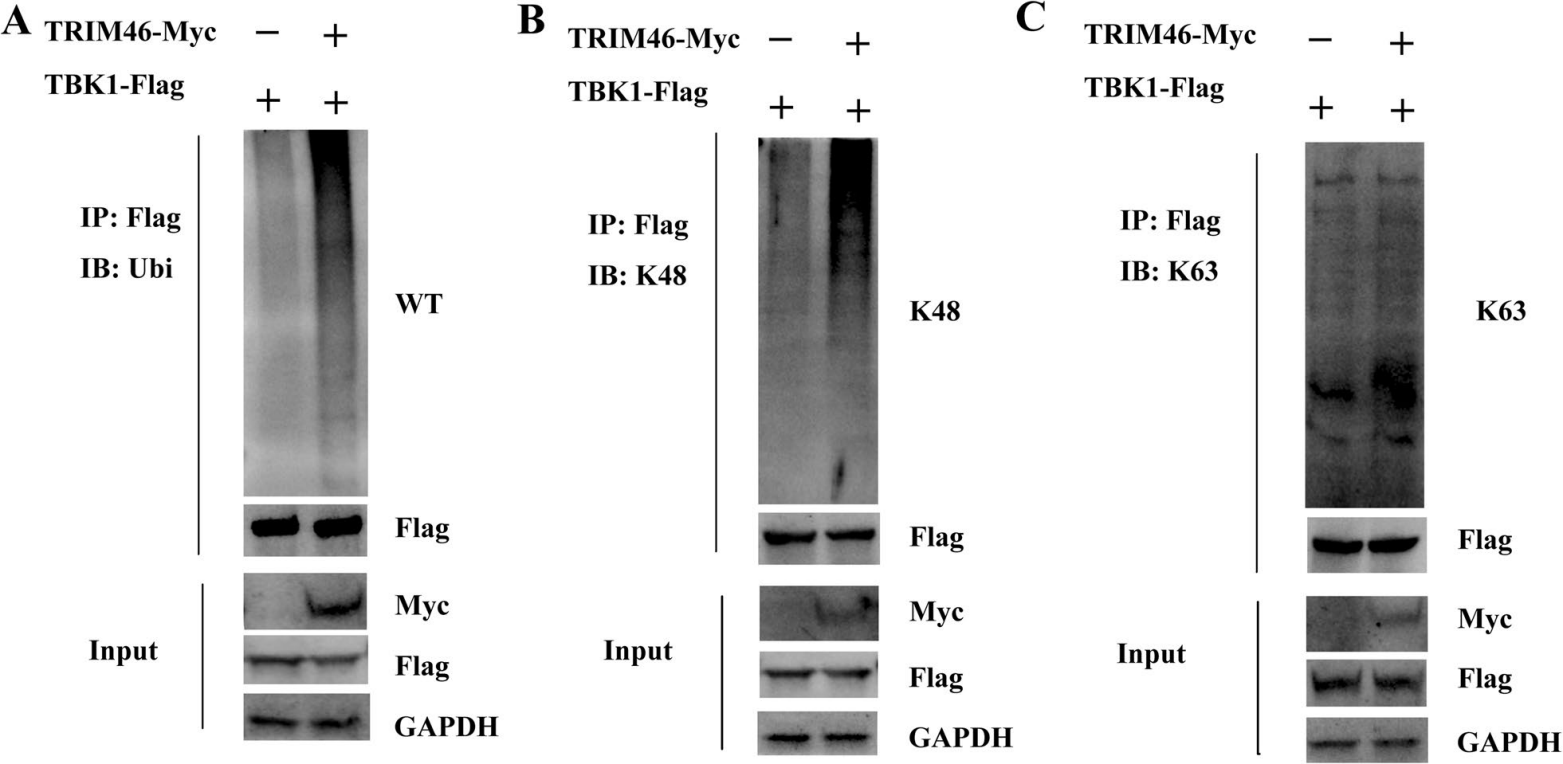
TRIM46 promotes K48-linked ubiquitination of TBK1. HEK293T cells were transfected with indicated plasmids for 24 h, after transfection, the cells were lysed and immune-precipitated with anti-Flag antibody and subjected to western blotting for detection of WT-linked (**A**), K48-linked (**B**), K63-linked (**C**) ubiquitination. Anti-Myc, anti-Flag and GAPDH were used for the input

To identify how TRIM46 regulates TBK1 expression, HEK293T cells were transfected with TBK1 together different doses of TRIM46 plasmids for 24 h transfection. The results showed that TRIM46 degraded TBK1 expression gradually (Additional file 1: Fig. S1B). Moreover, to investigate whether TRIM46 ubiquitinates TBK1, we transfected HEK293T cells with TBK1-Flag plasmids together with TRIM46-Myc or the empty vector for 24 h. The results demonstrated that overexpression of TRIM46 promoted TBK1 ubiquitination (Fig. 6A), suggesting TRIM46 promotes H7N9 replication by regulating TBK1-dependent innate immunity. To further determine whether TRIM46 ubiquitinates TBK1 via K48- or K63-linked ubiquitination, we determined K48-linked or K63-linked ubiquitination levels. The results demonstrated K48-linked ubiquitination of TBK1 mediated by TRIM46 (Fig. 6B), rather than K63-linked ubiquitination (Fig. 6C).

## Discussion

A number of studies have proposed that influenza virus could use multiple host cellular components to replicate and infect host cells. Besides, influenza has evolved to utilize host factors to inhibit the host innate immune response to evade immune surveillance and eradication [18–22]. For example, the influenza virus NS1 protein, which plays multiple roles between influenza virus and host innate immune responses, inhibits MAVS/IKK-mediated interferon production [23, 24]. Type I interferons play important roles in defending against virus replication, and virus infection induces a series of cellular antiviral signals to produce type I interferons [25, 26]. To screen and identify the host protein regulators involved in regulating the innate immune response against viruses would be helpful to identify therapeutic targets that manipulate the cellular antivirus responses. In the present study, we found that H7N9 virus-induced TRIM46 negatively regulated the production of type I IFNs by regulating the phosphorylation of IRF3. Furthermore, we discovered that TRIM46 interacts with TBK1, leading to TBK1 degradation via K48-linked ubiquitination. Our results suggest a novel function of TRIM46 in H7N9 virus infection.

TRIM proteins, belonging to the ubiquitin E3 ligase family, participate in regulating the host innate immune response against virus infection. A number of TRIM family proteins, such as TRIM22, TRIM25, TRIM35, TRIM56, have been found to be involved in the replication or pathogenesis of influenza virus [27–30]. TRIM proteins function as positive or negative regulators in host innate immune signaling pathways by mediating the ubiquitination of signaling protein [27–31]. For example, TRIM21 interacts with MAVS and catalyzes its K27-linked poly-ubiquitination to promote innate immune response against RNA viruses. By contrast, another TRIM family protein, TRIM29, inhibits host innate immunity by inducing K11-linked ubiquitination of MAVS [32, 33]. Our study demonstrated that TRIM46 promotes H7N9 virus infection by mediating the K48-linked ubiquitination of TBK1, which leads to TBK1 degradation, thus inhibiting innate immunity.

During virus infection, virus RNA is sensed by PRRs, which include RIG-I-like receptors (RLRs), NOD-like receptors (NLRs), and Toll-like receptors (TLRs) [34–37]. After influenza virus infection, influenza viral RNA is recognized by the RIG-I receptor, which activates and recruits the downstream TBK1/IKKγ/IKKε complex to induce IRF3 signaling, resulting in the production of type I interferons [38, 39]. Notably, viruses have involved multiple strategies to escape host innate immune surveillance and elimination, among which TBK1 is a target for virus-induced degradation. For instance, the SARS-CoV-2 membrane protein inhibits the production of type I interferons through induction of K48-linked ubiquitination of TBK1, which subsequently impairs IRF3 phosphorylation and dimerization [40]. Ubiquitin-conjugating enzyme 2S could interact with TBK1 and recruit USP15 to remove the K63-linked poly-ubiquitin chains of TBK1 [41]. Phosphatase PP4 dephosphorylates and deactivates TBK1 to inhibit the production of type I interferons [42]. The ubiquitin–proteasome pathway plays an important role in protein degradation, and ubiquitination of TBK1 is an important method of modulate the production of type I interferons during virus infection, during which process, viral proteins and host proteins participate [43–45]. In the present study, we found that TRIM46 could promote K48-linked ubiquitination of TBK1, which inhibited the phosphorylation of IRF3 and decreased the production of type I interferons.

## Conclusions

**Fig. 7:**
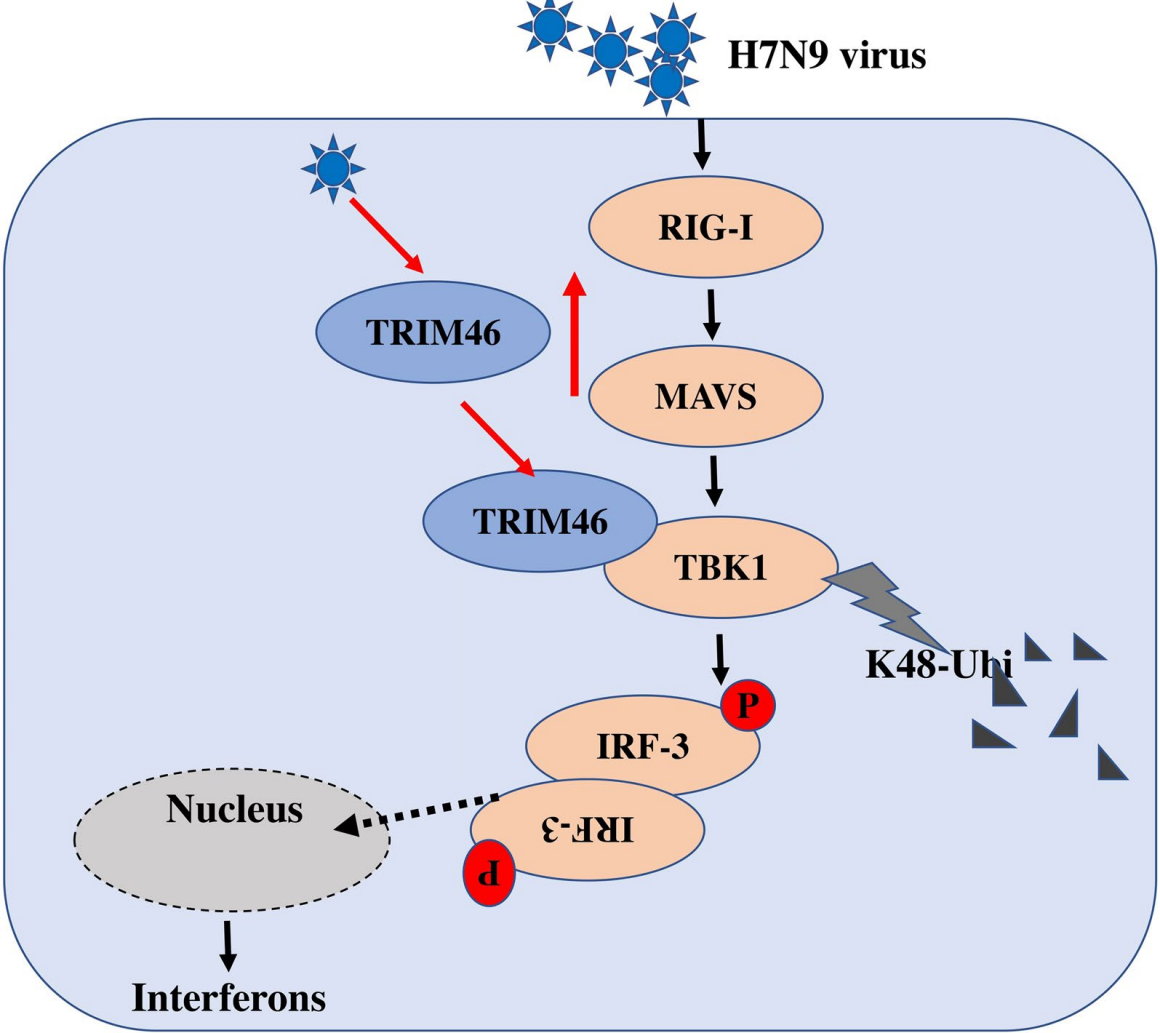
Schematic model of TRIM46-mediated K48-linked ubiquitination of TBK1. During avian influenza H7N9 virus infection, TRIM46 increased and interacted with TBK1. Then, TRIM46 mediated K48-linked ubiquitination of TBK1 and degraded TBK1, subsequently, decreased the phosphorylation of IRF3 and type I IFNs expression

Taken together, the results of the present our study show that H7N9-induced TRIM46 negatively regulates the production of type I interferons by inhibiting IRF3 phosphorylation by inducing K48-linked ubiquitination of TBK1 (Fig. 7). This study highlights the underlying mechanism by which H7N9 virus escapes the host innate immune response, which might lead to the development of novel antiviral agents to prevent or treat H7N9 virus infection.

## Supplementary Information

**Additional file 1.** Supplementary Fig 1. TRIM46 reduces TBK1 expression and interacts with TBK1 in HEK293T cells. (A) HEK293T cells were transfected with 1μg TBK1-Flag plasmids with 0 μg (-), 1μg (+) and 3 μg (++) TRIM46-Myc plasmids for 24 h, cells were lysed and subjected to western blotting to detect the expression of Flag tag and Myc tag, GAPDH was used as an internal control. (B) HEK293T cells were transfected with TRIM46-Myc plasmids with RIG-I-Flag, MAVS-Flag, TRAF3-Flag, TBK1-Flag and IRF3-Flag plasmids for 24 h. After transfection, cells were lysed and immuno-precipitated with anti-Flag antibody and subjected to western blotting to detect Flag and Myc tags. Input was detected and showed as Flag tag, Myc tag and GAPDH.

## Data Availability

All data generated or analysed during this study are included in this published article.
