# Supplementary figures and images for: Tripartite motif-containing protein 46 accelerates influenza A H7N9 virus infection by promoting K48-linked ubiquitination of TBK1
Source: Virol J. 2022 Nov 3;19:176. doi: 10.1186/s12985-022-01907-x (PMC9632593; doi:10.1186/s12985-022-01907-x)

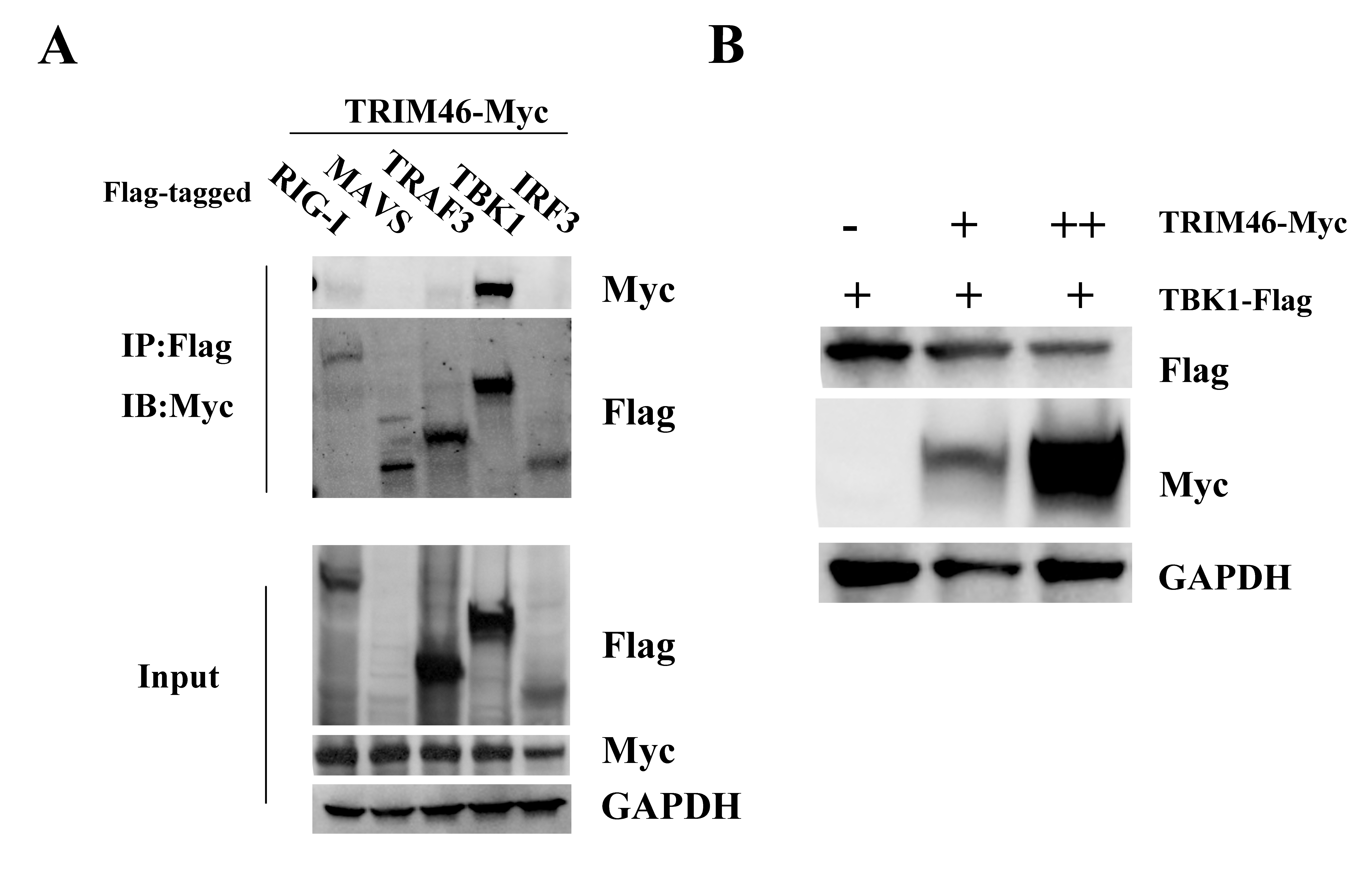

Supplement: Supplementary file 1 — Additional file 1. Supplementary Fig 1. TRIM46 reduces TBK1 expression and interacts with TBK1 in HEK293T cells. (A) HEK293T cells were transfected with 1μg TBK1-Flag plasmids with 0 μg (-), 1μg (+) and 3 μg (++) TRIM46-Myc plasmids for 24 h, cells were lysed and subjected to western blotting to detect the expression of Flag tag and Myc tag, GAPDH was used as an internal control. (B) HEK293T cells were transfected with TRIM46-Myc plasmids with RIG-I-Flag, MAVS-Flag, TRAF3-Flag, TBK1-Flag and IRF3-Flag plasmids for 24 h. After transfection, cells were lysed and immuno-precipitated with anti-Flag antibody and subjected to western blotting to detect Flag and Myc tags. Input was detected and showed as Flag tag, Myc tag and GAPDH. [file 12985_2022_1907_MOESM1_ESM.tif]
